# Exploring Spinal Cord Changes in Multiple Sclerosis Patients Using MRI

**DOI:** 10.3390/neurosci5010006

**Published:** 2024-03-12

**Authors:** Amani A. Alrehaili, Nahla L. Faizo, Batool M. Alsulimani, Raghad K. Alsulimani, Dana A. Aldwaila, Nada J. Alqarni, Nisreen Lutfi Faizo

**Affiliations:** 1 Department of Clinical Laboratory Sciences, College of Applied Medical Sciences, Taif University, P.O. Box 11099, Taif 21944, Saudi Arabia; 2 Department of Radiological Sciences, College of Applied Medical Sciences, Taif University, P.O. Box 11099, Taif 21944, Saudi Arabia; nfaizo@tu.edu.sa (N.L.F.); s44002952@students.tu.edu.sa (B.M.A.); s44002047@students.tu.edu.sa (R.K.A.); s44009573@students.tu.edu.sa (D.A.A.); s44000371@students.tu.edu.sa (N.J.A.); 3 Department of Clinical Anatomy, Faculty of Medicine, King Abdulaziz University, Jeddah 21589, Saudi Arabia; nlfaizo@kau.edu.sa

**Keywords:** central nervous system, magnetic resonance imaging, multiple sclerosis, spinal cord

## Abstract

Multiple sclerosis (MS) is an autoimmune disease affecting the central nervous system (CNS). The diagnosis of MS is based on clinical signs and symptoms as well as findings in magnetic resonance imaging (MRI) sequences by demonstrating the spatial and temporal dispersion of white matter lesions, which are thought to be typical of MS in distribution, shape, extent, and signal abnormalities. Spinal cord MRI can identify asymptomatic lesions and rule out malignancies or spinal stenosis in patients for whom brain imaging is not helpful in making an MS diagnosis. This study examines the MRI features of Saudi Arabian patients clinically proven to have MS with typical lesions exclusively evident in the spinal cord. This retrospective cross-sectional study was carried out in 151 patients who are confirmed cases of MS based on clinical findings and MRI results. Patients’ MRI data were reviewed from the picture archiving and communication system (PACS). The study revealed that MS incidence was higher in females than males and that the number of people diagnosed with MS increased in middle age. Cervical cord plaques and cervical cord curve straightening were the most frequent changes (67% and 56%, respectively), indicating that MRI can complement and even replace clinical data in MS diagnosis, leading to earlier, more precise diagnoses and speedier starts to treatment.

## 1. Introduction

Multiple sclerosis (MS) is a chronic autoimmune disease of the central nervous system (CNS) characterized by chronic inflammation, demyelination, gliosis, and neuronal loss. MS lesions occur at different times and in different CNS locations. For this reason, MS lesions are sometimes said to be “scattered in time and space” [1]. The definite etiology of MS is still not completely understood; however, different factors are thought to contribute to the disease occurrence. Environmental factors such as smoking and low vitamin D have shown significant association with MS [2]. In addition, people with genetic susceptibility are frequently affected, especially those with major histocompatibility complex (MHC) class II phenotype, human leukocyte antigen (HLA)-DR2, and HLA-DR4 [3]. Furthermore, MS can be brought on by diseases such as childhood chicken pox caused by viruses that have portions that resemble the myelin sheath and other viral infections that affect the immune system. Therefore, MS is thought to be caused by complex interactions between environmental variables and genetic vulnerability.

In the CNS, oligodendrocytes, which are a type of glial cells, generate myelin sheath which ensures efficient and fast nerve conduction. An inflammatory process involving T lymphocytes, B lymphocytes, and plasma cells is initiated around post-capillary venules and veins. This inflammatory reaction causes plaque formation which includes myelin loss, edema, and axon injury [4]. Perivenous demyelinated plaques expand to affect the normally appearing white matter to form the so-called Dawson fingers. Active tissue damage has been related to activated microglia and macrophages. As a result, neurodegenerative changes develop, affecting axons, neurons, and synapses [1]. When myelin, the protective membrane that insulates nerve fibers, is damaged and signals to and from the brain are disrupted, which causes a range of unpredictable symptoms [5]. While most people are able to regenerate damaged myelin in the CNS innately, patients with MS lose their ability to do so for unknown reasons [6]. Neurologic symptoms vary depending on the site of the lesion and may include visual disturbances, numbness and tingling, local weakness, bladder and bowel incontinence, and cognitive dysfunction [4]. Thus, MS symptoms, severity, and degree of disability are all highly variable and unpredictable [7]. In the majority of cases, people experience periods of comparatively good health interspersed with intervals of deteriorating symptoms; however, the disease generally worsens over time. MS is most commonly diagnosed between ages 20 and 50, and women are twice as likely as men to develop it.

The fundamentals of MS diagnosis rely on the presence of CNS symptoms and signs as well as radiological magnetic resonance imaging (MRI) findings demonstrating the spatial and temporal dispersion of white matter (WM) lesions. Over the last three decades, the technical innovation of MRI methods has improved dramatically, yielding a significant impact in the diagnosis and follow up of MS [8]. Today, with the continuous advancement in MRI techniques, MRI has become a useful radiological technique to elucidate neuroinflammatory and autoimmune mechanisms. WM lesions, which are considered typical of MS in distribution, morphology, and extension, demonstrate signal abnormalities on MRI sequences such as T1-weighted prior to or after gadolinium contrast administration, T2-weighted fluid-attenuated inversion recovery (FLAIR) or short-tau inversion recovery (STIR), and diffusion weighted pulse sequences. 

Despite the typical presentation of MS, asymptomatic or subclinical cases are discovered incidentally by imaging. Thus, the most accurate way to detect the asymptomatic spread of lesions over time and space is with MRI [9], which can support and even replace clinical information in identifying cases of MS, allowing for earlier and more accurate diagnoses and treatment [10]. When used in the proper clinical context, the pattern and progression of WM lesions have made MRI anomalies an essential diagnostic tool for early MS diagnosis. The first crucial function of MRI in MS diagnosis is to enable an early diagnosis using the international panel diagnostic criteria for patients with cancer in situ, which includes MRI for dissemination in space and time. Within the first year following a single attack, there is 94% sensitivity and 83% specificity for diagnosing MS [9]. Although conventional MRI has a distinguished diagnostic sensitivity, efforts to seek high specificity to MS are still in progress [11].

For brain MRIs, a magnetic field intensity of at least 1.5 T is required, but 3 T MRIs are recommended because of their enhanced image resolution and signal-to-noise ratio, which increase their sensitivity to focal MS lesions [12,13]. When compared to the standard field strength of 1.5 T, higher magnetic field strengths like 3 T have been shown to exhibit improved sensitivity for WM and grey matter lesions in MS patients and clinically isolated syndrome, but this has no bearing on the possibility of an earlier MS diagnosis [13,14]. If brain imaging is not helpful in diagnosing patients suspected of having MS, spinal cord imaging can be used to rule out spinal stenosis or tumors and to identify asymptomatic lesions. When used in the proper clinical setting and only when other diagnoses have been ruled out, MRI evidence can be helpful in arriving at a clinical diagnosis of MS [9].

The enormous promise of MRI for tracking MS patients’ condition and course of treatment has been eclipsed to some extent by its diagnostic function regarding the condition. However, there has been a significant increase in understanding in this area in recent years, especially in relation to different MRI techniques and the idea of therapy efficacy and safety monitoring prediction [10]. As a result, more practitioners are realizing and appreciating the potential of MRI measurements in determining and tracking therapy efficacy. The use of MRI in treatment monitoring has expanded with the release of a new generation of more effective MS medicines [15]. Furthermore, new imaging techniques are needed to track disease activity in light of the introduction of immunomodulating medications, which concentrate on other pharmacodynamic pathways (such as remyelination) to stop the progression of the illness [10]. Regrettably, the practical clinical application of these sophisticated MRI techniques is still restricted due to institutional variations in hardware availability, scan procedures, and other technical factors [16].

A systematic review in 2023 by Nathoo et al. assessed the variations in MRI findings among people with MS of different racial and cultural backgrounds, such as African American and Latin American populations in the United States, Japan, the Middle East, and Ethiopia; only two studies examined MS in the Middle East [13]. One compared the features of relapsing remitting MS in Medina, Saudi Arabia (KSA), and Edmonton, Canada; more Caucasian MS patients in Canada exhibited spinal cord lesions and posterior fossa lesions than Bedouin Arabs [12,13]. According to the authors, this may be explained by Canada’s greater rate of spinal cord imaging [14,17]. Although the Caucasian Canadian MS patients had more infratentorial and spinal cord lesions, the KSA population had higher Expanded Disability Status Scale (EDSS) scores [14,17]. The other study examined patients with MS who had just received a diagnosis and were visiting a tertiary hospital in Doha, Qatar [12,13], 68% of whom were Asian (categorized as “other”) and 31% of whom were non-Qatari Arabs. In the group of Arabs followed up for 36 months, 20% saw a decline in EDSS scores and 55% showed signs of radiological disease development. It was not possible to compare the clinical and radiological activity of the subgroups of Qataris, non-Qatari Arabs, and Asians (“other”) [14,18].

Only limited research has been performed regarding MS symptoms in KSA. In order to characterize the clinical features and demographics of 82 MS patients at the Armed Forces Hospital in Khamis Mushayt and the Aseer Central Hospital in Abha, a retrospective study was recently carried out [19]. It was found that the majority of the research cases (37.8%) had ocular symptoms when they first appeared. However, Heydarpour et al.’s systematic study and meta-analysis of MS epidemiology revealed that the most common symptom among Saudi MS patients was weakness [20]. These outcomes support those of a cross-sectional study conducted in KSA by AlJumah et al.; the researchers discovered that, of the 2516 MS cases, muscle weakness was the most common symptom at the onset (57.1%), followed by visual symptoms (48.2%) [21]. 

Overall, very little research has been conducted with Saudi MS patients and most studies that have been carried out focus on EDSS scores or symptoms related to MS. None of those studies examined spinal cord changes in patients with MS using MRI. In addition, MS is now more common in KSA than it was just a few years ago [19,21]. In 2015, KSA launched its first national MS registry involving multiple centers; it now has 20 participating hospitals. According to estimates, the nationwide prevalence of MS in KSA in 2018 was 40.40/100,000 overall and 61.95/100,000 among Saudi citizens [22]. The present study analyzes the spinal MRI characteristics of clinically confirmed Saudi MS patients.

## 2. Materials and Methods

A retrospective cross-sectional study was conducted at Alhada Armed Forces Hospital and King Abdulaziz Specialist Hospital in the Taif region from December 2022 to May 2023. The study received approval at 20.FBR.2023from the the Research Committee of the Ministry of Health (Ref: HAP-02-T-067), and the research and Ethics Committee of the Ministry of Defense at Western Region (Ref: REC. 2023-729).This study included data from 151 MS confirmed cases (47 males and 104 females). Their diagnoses were based on the history and MRI findings on the picture archiving and communication system (PACS).

The following inclusion criteria were used in this study: (1) Saudi individuals whose clinical symptoms and MS-related indicators led to an MS diagnosis; (2) while exclusion criteria included individuals who did not have brain MRIs, patients were recommended for brain MRI. Using PACS, patient data were examined and tables containing the following data were created: age, gender, and MRI results (cervical spine plaques, straightening of cervical curve, disc herniation, thoracic spine plaques, disc degeneration, spinal cord atrophy, straightening of lumbar curve, spinal canal stenosis, and spinal cord swelling). All the spinal MRIs were reviewed and reported by an expert radiologist. The patients were also divided into five age groups (18–24, 25–34, 35–44, 45–54, and 55–64 years old). All reviewed patients underwent spinal MRI in a 1.5 T scanner utilizing a spinal phased array coil with the following parameters: sagittal T1W and T2W fast spin echo sequence, field of view 24 × 24 cm, matrix size 256 × 256, slice thickness 3 mm, repetition time 9000 ms, echo time 100 ms, number of signal averages, and scan time around 20 min. Subject confidentiality was maintained in a deliberate manner during data collection and reporting. 

### Statistical Analysis

Data were revised, coded, entered, tabulated, and analyzed using SPSS v. 20. The countable data such as demographic and spinal MRI changes were represented in frequency and percentage, while measured data were tested by the *t*-test and ANOVA test which were used to compare categorical variables. Statistical significance was assigned to any *p*-value equal to or below 0.05.

## 3. Results

**Figure 1:**
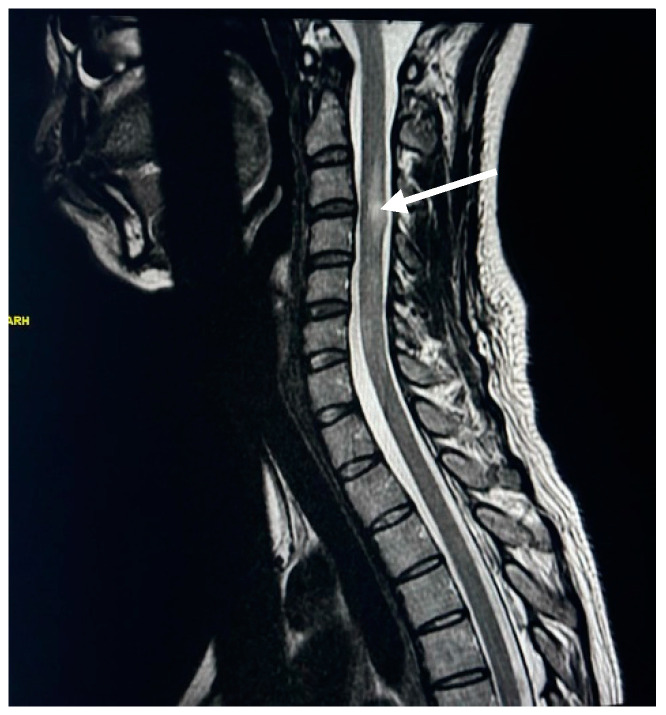
T2W MR image of cervical spine plaque. The white arrow demonstrates a high signal intensity representing cervical plaque.

**Figure 2:**
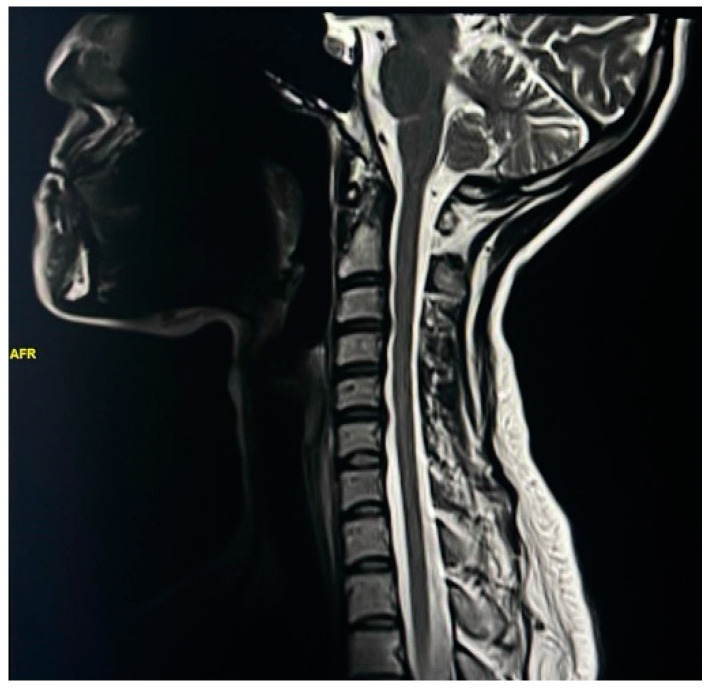
T2W MR image of cervical curve straightening. The image demonstrates straightening of the cervical spine curvature of a 34-year-old male patient with MS.

**Figure 3:**
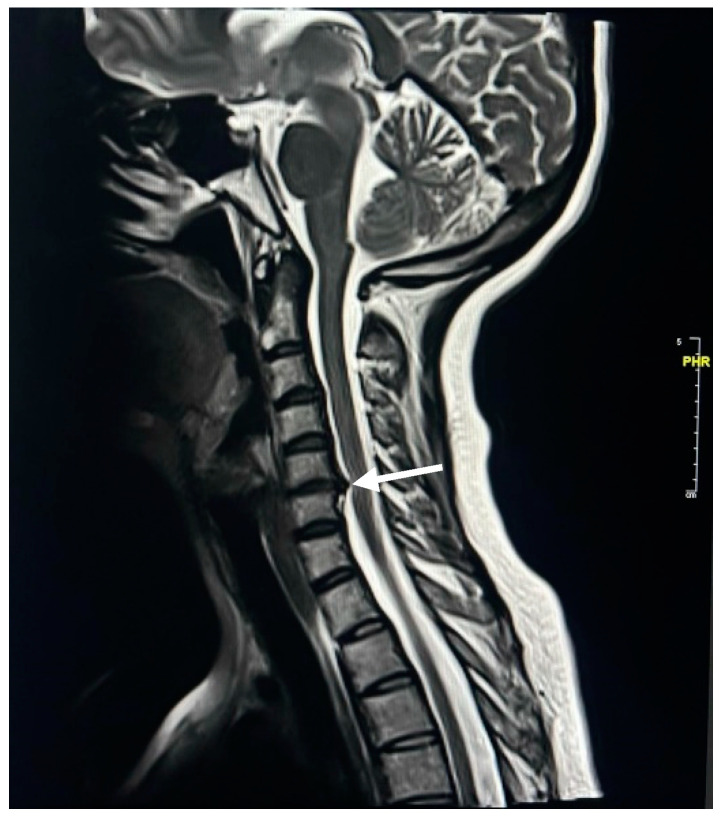
T2W MR image of disc herniation. The white arrow demonstrates disc herniation in the 5th intervertebral disc of a 46-year-old female with MS.

**Figure 4:**
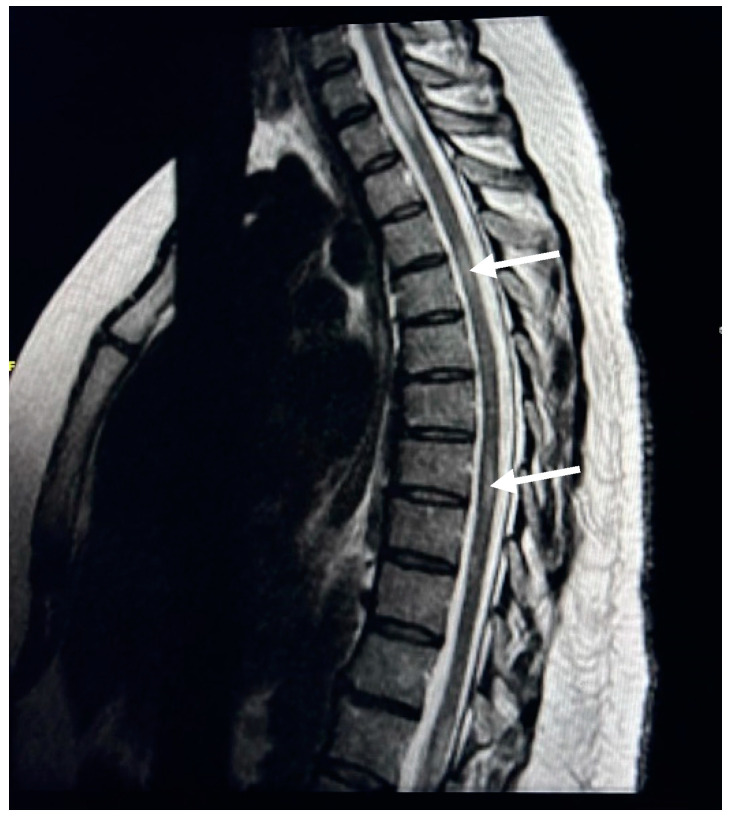
T2W MR image of the thoracic spine. This image illustrates high signal intensities (white arrows) in the thoracic spine of a 31-year-old female.

**Figure 5:**
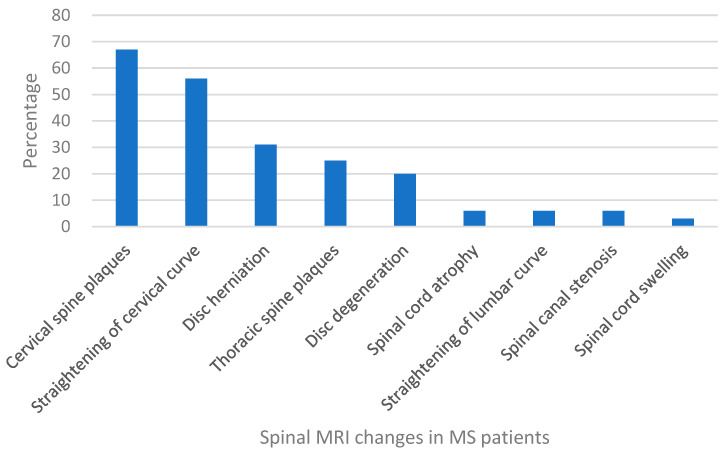
Spinal MRI changes in MS patients.

Spinal MRI changes in MS patients were examined and analyzed. The results showed that cervical spine plaques were most present in 67% of cases (Figure 1), followed by straightening of cervical curve in 56% of patients (Figure 2), disc herniation in 31% of patients (Figure 3), thoracic spine plaques in 25% of patients (Figure 4), and disc degeneration in 20% of patients. Less commonly (≤6%), changes in lumber curve straightening, spinal cord atrophy, swelling, and stenosis were identified (Figure 5).

Gender differences for each spinal cord change in MS patients were also evaluated. The results showed that the percentage of females was higher than the percentage of males with an overall statistically significant difference (*p*-value = 0.032, Table 1). For example, cervical spine plaques occurred in 46.36% of females and 21.19% of males, while straightening of cervical curves occurred in 44.37% of females and 13.91% of males and disc herniation occurred in 21.85% of females and 9.27% of males, except for straightening of lumbar curve and spinal canal stenosis in which males were slightly higher than in females as shown in Table 1.

One-way ANOVA was used to compare spinal cord changes among different age groups and results showed a significant difference with *p*-value < 0.05 (Table 2). For the most common spinal cord change, cervical spine plaques, the results showed that the most affected age group consisted of patients aged 25–34 years (~27%), followed by those aged 35–44 years (~18%); the percentage of patients in whom plaques appeared in the rest of the age cohorts was less than 15%, as shown in Table 2.

Similar results were observed for straightening of cervical curve, the most frequently affected age group was 25–34 years (around 25%), as shown in Table 2. For associated disc herniation and thoracic spine plaques, the 25–34 age group was most affected (about 11%). Regarding disc degeneration, the most affected age group was 45–54 years old (~7%). For lumbar curve straightening, spinal cord atrophy, and swelling, the two age groups 25–34 and 35–44 years were affected equally (see Table 2 for full results).

## 4. Discussion

MS is a chronic demyelinating and neurodegenerative disease that affects the CNS and is mediated by lymphocytes which infiltrate the CNS through the blood–brain barrier (BBB) causing inflammation, demyelination, and axonal injury. Early in the course of MS, patients present with focal demyelinating plaques, decreased oligodendrocytes, gliosis, and infiltration of lymphocytes and macrophages. In later stages, MS causes atrophy of the white and gray matter, inflammation, microglia activation, and diffuse of the injury to the normal-appearing white matter [23]. MRI abnormalities have become an essential diagnostic tool for early MS diagnosis due to the pattern and progression of MRI lesions in the right clinical scenario. When a lesion spreads asymptomatically over time and space, MRI is the most sensitive technique for detecting it [12], as MRI is routinely used in clinical practice to detect and monitor inflammatory lesions in patients with MS [19]. Correlative studies have shown that MRI is sensitive to pathological features of MS which include inflammation, demyelination, and axonal injury [24]. The present study has evaluated spinal cord changes that occur due to MS using spine MRI scans in Saudi patients.

Interestingly, focal spinal cord lesions have been found in about one-third of patients presenting with symptoms of early demyelinating disease without displaying any spinal cord symptoms [25]. Our results reveal that the key changes that appeared in MRI of the MS confirmed cases included cervical spine plaques, straightening of cervical curve, disc herniation, thoracic spine plaques, disc degeneration, spinal cord atrophy, straightening of lumbar curve, spinal canal stenosis, and spinal cord swelling. In addition, the study clearly confirms the prevalence of MS in females over males by a ratio of roughly two to one and that it most often emerges between ages 20 and 50 [22,26].

Although there are no definite reasons for why females are more affected with MS than males, there are some possibilities of genetic, hormonal, immunologic, and environmental interactions [2,3]. For example, the indoor lifestyle for female is well-known in Saudi Arabia and women are fully covered when they are outdoor with traditional clothing. This minimizes their exposure to sun and makes them more vulnerable to vitamin D deficiency which shows a strong association with MS [2].

The results also reveal that the most common spinal cord change in people with MS is plaques in the cervical spine cord. This result accords with the findings of a previous study which showed that spinal cord lesions were continuously observed in 80–90% of MS patients and that most MS plaques were in the cervical spine cord. The loss of myelin forms scar tissue called plaques, which entail damage to the myelin that protects nerve cells and appear in MRI as multiple hyperintense lesions in different regions of the brain and spinal cord [24]. Our findings show that plaques in the cervical spinal cord were observed in 67% of patients, while plaques in the thoracic spinal cord were found in 25% of patients, which accords with another spinal cord lesion study that found MS plaques in the cervical spinal cord in 59% of patients and MS plaques in the lower thoracic spinal cord (T7–T12) of 20% of participants [26]. This indicates that cervical spine plaques are most prevalent in Saudi MS patients. Our study adds that the most affected age group is 35–44 years, at 70% for female and 30% for male patients.

The study demonstrates that the second most common spinal cord change is cervical straightening. The overall percentage of straightening of cervical curve is 58% (44% for females and almost 14% for males) and the most common age group is 25–34 years. This result accords with the finding of a study by Fernandez et al. that muscle spasms, which can lead to pain and cramping, are one of the most common changes in MS, although the etiology of muscle spasms in MS patients results in considerable clinical heterogeneity [27]. It is unclear why cervical straightening occurs in MS patients; however, it could be explained by the spondylotic changes that may accompany the disease. Another possible etiology of straightening is spinal cord injury, such as a herniated disc or spinal canal stenosis, and MS plaques may cause acute episodes of low back pain. A disc may herniate plaques and compress a nearby spinal nerve root, causing irritation and inflammation [28]. 

In addition, there is evidence linking MS to degenerative disc disease [29]. Abnormalities in the spinal cord MRI signals, most typically in the cervical cord, have been observed in up to 90% of MS patients [30]. Concurrently, new research indicates that MS patients may experience degenerative changes in their cervical spine more frequently [31,32]. In order to examine the incidence and clinical impact of herniated discs in patients with MS, the hospital documentation of patients treated medically for symptomatic cervical and lumbar disc herniation between 2020 and 2023 to determine the rate of comorbid disc herniation in MS patients was evaluated. The results showed that the number of individuals diagnosed with symptomatic cervical and lumbar disc herniations increased by 34% between the ages of 25 and 34 years, and the incidence of these conditions was higher in women (68%) than in men (32%).

Furthermore, this study indicates that the incidence of disc degeneration was observed in almost 14% of females and 6% of males diagnosed with MS. These results are consistent with those of Drenska et al. [31], who demonstrated that disc herniation was found in 19.4% of MS patients and that women were affected more than men by a ratio of two to one. In addition, the results of the present study accord with the findings of an extensive prospective cohort study of Japanese adults, where cervical disc degeneration disease was present in 26.3% of men and 27.9% of women aged 21–49 years [32]. Moreover, a recent study by Omid-Fard et al. on 80 young MS patients revealed that most patients demonstrated cervical disc degeneration which was independent of spinal cord lesions, a phenomenon that should be further investigated [28].

Spinal cord atrophy is one of the long-term sequelae of MS, especially in its most disabling forms, and the cervical spinal cord is most affected [33]. Casserly and his colleagues demonstrated that the magnitude of spinal cord atrophy is greater in progressive MS than in relapsing cases and correlated with clinical disability [34]. It might even be detected four years prior to conversion to progressive MS [35]. The primary cause is thought to be Wallerian degeneration due to changes occurring in the brain, not tissue loss due to primary spinal cord disease [31]. Although brain MRI is used in investigating all MS patients, spinal cord MRI is recommended in certain conditions such as (1) clinical suggestion of spinal cord lesion or primary progressive MS, (2) normal brain MRI in patients with MS clinical presentation, and (3) inconclusive brain MRI [26].

The present study shows that the percentage incidence of straightening of lumbar curve is 6% and spinal canal stenosis is almost 4% with a slightly higher impact on males than on females. These findings are surprising since MS and its associated changes are more common in females than in males. However, this could be related to the fact that neurodegeneration occurs more noticeably in males than in females [36]. Cervical spine spondylosis, which might be the cause of spinal stenosis, has been reported in MS patients of younger age and has been positively correlated with increased brain lesions and disability [37]. It is still unclear whether spinal canal stenosis is an exacerbating factor of MS or MS-related spasticity is a leading cause of spinal degenerative disease. 

Another change seen in some patients with MS is swelling of the spinal cord; this appeared in almost 5% of cases with the presence of cervical and thoracic MS plaques, where the first three age groups were mostly affected. A previous study of spinal cord swelling in patients with MS showed that it was rarely reported; the authors of that study suggest a possible cause: MS plaques may become swollen in acute stages due to edema [38].

Although it in known than MS can be presented with dispersion in time and space, yet the differences in finding among different patients in our study could be related to the scanner field strength. All cases studied in this research were scanned in 1.5 T. It was previously proposed that 3 T would improve the detection of brain and spinal MS lesions [39]. In this regard, a multicenter study by Hagens et.al. compared the effect of high field strength (3 T) and 1.5 T in detecting brain and spinal MS lesions. The study revealed the significant improvement in MS brain lesion detection at 3 T when compared to 1.5 T especially in T2-weighting images. However, high field strength has no improved effects on the identification of spinal cord lesions. As there was no added value in using 3 T over 1.5 T for identifying spinal MS lesions, the potential clinical significance of further enhancing the signal-to-noise ratio and increasing resolution remains unclear. 

Overall, the most commonly found changes in spinal cord using MRI were observed in MS patients between 25 and 54 years, suggesting that spinal cord changes vary and are generally more predominant in women than in men. These findings support previously published data specifying that MS most commonly emerges between the ages of 20 and 50, women are twice as likely as men to develop it, and MS symptoms, severity, and degree of disability are all highly variable and unpredictable [7].

## 5. Conclusions

The MRI reports of 151 Saudi MS patients between 2020 and 2023 were included; they showed that the number of individuals diagnosed with MS increased in middle age and that its incidence was higher in women than men. The most common changes were cervical cord plaques and straightening of the cervical cord curve. This study reinforces the use of MRI to support the clinical information in the diagnosis of MS, allowing for earlier and more accurate detection and a more rapid start to treatment.

The fact that this study was limited to two large hospitals in the city of Taif means that it may not be generalizable to all MS patients in Saudi Arabia. Thus, future studies of spinal cord changes in MS patients should encompass the country as a whole to offer a more detailed account of clinical changes and manifestations in Saudi MS patients. Furthermore, the study supports the need for establishing guidelines and recommendations to standardize and explain doctors’ decision-making options when diagnosing MS patients, given the increasing incidence of MS in Saudi Arabia and the development of innovative alternative medications. As the diagnosis of MS depends heavily on imaging, specifically MRI, evidence-based radiology-related guidelines are particularly important for managing the condition. 

## Figures and Tables

**Table 1:** Analysis of changes in the spinal cord in MS patients based on gender.

Spinal Cord Change	Female		Male	
	No.	%	No.	%
Cervical spine plaques	70	46.36	32.00	21.19
Straightening of cervical curve	67	44.37	21.00	13.91
Disc herniation	33	21.85	14.00	9.27
Thoracic spine plaques	28	18.54	10.00	6.62
Disc degeneration	21	13.91	9.00	5.96
Straightening of lumbar curve	4	2.65	5.00	3.31
Spinal cord atrophy	4	2.65	4.00	2.65
Spinal canal stenosis	3	1.99	4.00	2.65
Spinal canal swelling	4	2.65	1.00	0.66
	104		47	
*p*-value	0.0328			

**Table 2:** Analysis of spinal cord changes in MS patients based on age.

Spinal Cord Change		Age Cohorts					
		18–24	25–34	35–44	45–54	55–64	Total
Cervical spine plaques	No.	10	41	27	19	5	102
	%	6.62	27.15	17.88	12.58	3.31	67.55
Straightening of cervical curve	No.	9	38	21	17	3	88
	%	5.96	25.17	13.91	11.26	1.99	58.28
Disc herniation	No.	3	17	9	13	5	47
	%	1.99	11.26	5.96	8.61	3.31	31.13
Thoracic spine plaques	No.	2	18	9	6	3	38
	%	1.32	11.92	5.96	3.97	1.99	25.17
Disc degeneration	No.	0	7	7	11	5	30
	%	0.00	4.64	4.64	7.28	3.31	19.87
Straightening of lumber curve	No.	0	3	3	2	1	9
	%	0.00	1.99	1.99	1.32	0.66	5.96
Spinal cord atrophy	No.	1	2	2	1	2	8
	%	0.66	1.32	1.32	0.66	1.32	5.30
Spinal canal stenosis	No.	1	3	0	2	1	7
	%	0.66	1.99	0.00	1.32	0.66	4.64
Spinal cord swelling	No.	1	2	2	0	0	5
	%	0.66	1.32	1.32	0.00	0.00	3.31
*p*-value	0.045						

## Data Availability

The raw data supporting the conclusions of this article may be available by the authors (contact authors at nfaizo@tu.edu.sa) upon request after application to Alhada Armed Forces Hospitals and the Ministry of Health Hospitals in the Taif region. Taif region is Saudi Arabian Military region and thus, sharing the data for this research requires approval.
